# The proportion of tumour cells is an independent predictor for survival in colorectal cancer patients

**DOI:** 10.1038/sj.bjc.6605674

**Published:** 2010-04-20

**Authors:** N P West, M Dattani, P McShane, G Hutchins, J Grabsch, W Mueller, D Treanor, P Quirke, H Grabsch

**Affiliations:** 1 Pathology and Tumour Biology, Leeds Institute of Molecular Medicine, University of Leeds, St James′s University Hospital, Leeds LS9 7TF, UK; 2 Centre for Epidemiology and Biostatistics, Institute of Genetics, Health and Therapeutics, University of Leeds, Leeds LS2 9JT, UK; 3 Gemeinschaftspraxis Pathologie, Starnberg, Germany

**Keywords:** colorectal cancer, proportion of tumour, point counting, virtual slides

## Abstract

**Background:**

The proportion of epithelial and stromal cells in tumours is thought to have an important role in the progression of epithelial malignancy. We aimed to determine whether the relative proportion of tumour (PoT) was related to survival in colorectal cancer.

**Methods:**

The PoT at the luminal surface was measured by point counting using virtual tissue sections in a series of 145 colorectal cancer cases. The relationship of PoT to clinicopathological parameters including cancer-specific survival was analysed. Modified receiver operating characteristic curves were used to determine the optimum cut off points to dichotomise the data for survival analyses.

**Results:**

Tumours with PoT-low (⩽47%) were associated with significantly lower cancer-specific survival when compared to PoT-high (hazard ratio (HR)=2.087, 95% CI=1.088–4.003, *P*=0.024). On sub-analysis, the prognostic effect remained significant in colonic tumours (HR=2.474, 95% CI=1.132–5.408, *P*=0.019) and tumour, node, metastasis stage III disease (HR=3.480, 95% CI=0.325–9.136, *P*=0.007). Multivariate Cox regression analysis demonstrated that PoT was an independent prognostic marker when adjusted for age, T stage, N stage and extramural vascular invasion (*P*=0.017).

**Conclusion:**

This study suggests that a low proportion of tumour cells in colorectal cancer is related to poor cancer-specific survival. A relatively quick, inexpensive and well-established method such as point counting on diagnostic tissue sections could be used to identify a subset of patients who may benefit from adjuvant therapy.

Colorectal cancer (CRC) is a common disease and despite significant advances in its treatment, the 5-year overall survival remains around 50% (Cancer Research UK, 2009). Recent figures show that CRC is now the second commonest cause of cancer related mortality in the United Kingdom with over 16 000 deaths occurring annually (Cancer Research UK, 2009). Staging systems such as Dukes (Gabriel *et al*, 1935) and tumour, node, metastasis (TNM) (Sobin and Wittekind, 1997, pp 66–69) are routinely used to predict prognosis following surgery, however, patients diagnosed at the same stage of disease often have markedly different outcomes (Morris *et al*, 2007). Current research aims to identify additional prognostic markers that can be used to stratify CRC patients and identify those which may benefit from adjuvant therapy.

Similar to all other malignant epithelial tumours, CRC is composed of carcinoma cells admixed with stromal fibroblasts, lymphatic and vascular channels, and inflammatory cells, often referred to as the tumour microenvironment. This microenvironment is becoming increasingly recognised as having an important role in tumour cell invasion and the ability to metastasise (De Wever and Mareel, 2003).

Very few studies in selected cancer subtypes, such as breast (Baak *et al*, 1985), lung (Nakajima, 1991; Maeshima *et al*, 2002; Das Neves Pereira *et al*, 2004), skin (Breuninger *et al*, 1997) and prostate cancer (Yanagisawa *et al*, 2007) have quantified the cellular components of primary tumours and demonstrated that the tumour composition is associated with patient survival. In CRC, the number of stromal myofibroblasts (Tsujino *et al*, 2007), vimentin expression (Ngan *et al*, 2007) and degree of stromal desmoplasia (Halvorsen and Seim, 1989; Shepherd *et al*, 1997; Sis *et al*, 2005) have been associated with patient prognosis in the past. So far, only one study has suggested that the proportion of tumour cells in CRC may be important (Mesker *et al*, 2007). However, this result was based upon a relatively small number of cases using qualitative visual estimation of the epithelial component rather than measuring the components objectively by a well-established morphometric method, such as point counting that was first described in the 1940s (Chalkley, 1943) and developed further by Weibel in the 1960s and 70s (Weibel, 1969).

We hypothesised that the relative proportion of tumour (PoT) determined by objective point counting on virtual (scanned) haematoxylin and eosin-stained slides is related to cancer-specific survival in CRC patients.

## Materials and methods

### Patients

A total of 145 patients who had potentially curative resections for colorectal adenocarcinoma at the Marienhospital, Düsseldorf, Germany between January 1990 and December 1995 were selected for this study. None of the patients had received pre-operative chemotherapy or radiotherapy. The median follow-up time was 4.3 years (interquartile range=2.2–6.2 years) and 107 patients (73.8%) were alive at the end of the study period.

### Clinicopathological data

Histopathological staging data was obtained from the pathology reports or from slide review by one of the pathologists (WM) and included the site of the tumour, extramural vascular invasion status, maximum depth of invasion (pT), lymph node involvement (pN) and distant metastasis (pM) according to TNM classification, 5th edition (Sobin and Wittekind, 1997, pp 66–69). In addition, we had access to data regarding patient age at diagnosis and whether or not adjuvant therapy was given.

### Measurement of the relative PoT

**Figure 1:**
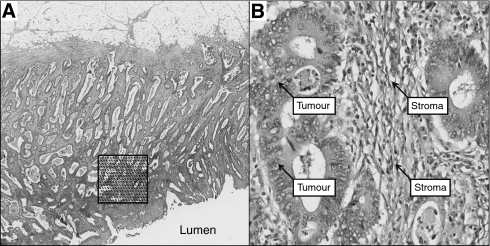
Morphometric method for establishing the PoT. (**A**) Selection of a 9 mm^2^ area at the luminal surface of a haematoxylin and eosin-stained representative section of colorectal cancer. A total of 300 points are randomly inserted into the selected area. (**B**) Annotation of four individual points comprised of tumour and stroma.

Four μm thick haematoxylin and eosin-stained tissue sections prepared according to standard protocols from one tumour block representing the deepest tumour infiltration into the wall and the largest tumour volume were scanned at × 40 magnification with an automated scanning system (Aperio XT, Aperio Technologies, Vista, CA, USA). Using a digital slide viewer (ImageScope v8.0, Aperio Technologies), slides were inspected after scanning and a number of preliminary analyses were carried out on a small number of cases to establish the optimal frequency of points and optimal size of the area required to accurately assess the PoT (data not shown). An area of 9 mm^2^ was selected from the luminal surface of each case in which the tumour cell density seemed to be greatest after visual inspection. A grid with a systematic random sample of 300 points was then superimposed on the selected area using newly developed virtual graticule software (RandomSpot, University of Leeds, Leeds, UK) to count the number of times the point fell over each of the categories. Large areas of necrosis and mucus at the surface were avoided when selecting the area. The number of necessary measurement points established in our preliminary work was consistent with previous descriptions of the number of points needed to accurately assess PoT (Baak *et al*, 1991, pp 189–209). The following categories were used in the scoring system: tumour (the point falls onto a viable cancer cell), stroma, tumour lumen, necrosis, vessel, inflammation and non-informative (unclassifiable). One of the authors (MD) was trained by an experienced pathologist (HG) in recognising the different categories and subsequently navigated through each point and categorised the material underneath the point while blinded to the histopathological and survival data (Figure 1). To assess interobserver variation, a random sample of 40 cases were double scored by a second pathologist (GH). As MD and GH agreed in 98.3% of the counts (*κ*=0.971), double scoring of all counts was felt to be unnecessary. The PoT was expressed as a percentage fraction of all the informative points per case. Each case took approximately 20 min to score.

### Statistical analyses

Statistical analyses were performed using the Statistical Package for the Social Sciences (SPSS v15.0, Chicago, IL, USA). Comparisons between PoT and clinicopathological variables were performed using the Mann–Whitney *U* or Kruskal–Wallis test as appropriate. Correlation analyses were performed using Pearson's correlation coefficients.

**Figure 2:**
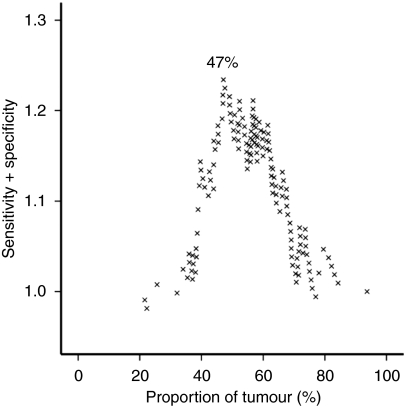
Modified receiver operating characteristic curve used to determine the optimal cut off point for PoT for the subsequent survival analyses. The optimal point is determined from the peak of the curve.

The primary endpoint was death attributable to CRC. Cancer-specific survival data were available for all patients. Using a modified receiver operating characteristic curve approach developed by one of the authors (PMcS), the cut off for dichotomisation of PoT with the highest sensitivity and specificity regarding survival prediction was calculated (Figure 2). Using this approach, PoT was classified as either PoT-high (>47% of tumour cells within the tumour) or PoT-low (⩽47% of tumour cells within the tumour).

Patients who died within 30 days after surgery (post-operative mortality) were excluded from the study. Univariate survival analyses were performed using Kaplan–Meier curves (Kaplan and Meier, 1958) and differences between the groups assessed with the log-rank test. To assess whether the potential new prognostic marker predicts survival independent from known prognostic markers, such as pT, pN, extramural blood vessel invasion and age, multivariate survival analyses were performed using the Cox proportional hazards regression model (Cox, 1972). *P-*values of less than 0.05 were considered to be statistically significant.

### Ethical approval

Ethical approval for the study was granted by the Northern and Yorkshire Research Ethics Committee, Jarrow, UK (unique reference number 08/H0903/62).

## Results

### Clinicopathological data

The median age of patients was 69 years (interquartile range from 61 to 76 years). The remaining clinicopathological data is displayed in Table 1.

### Relative proportion of tumour (PoT)

**Figure 3:**
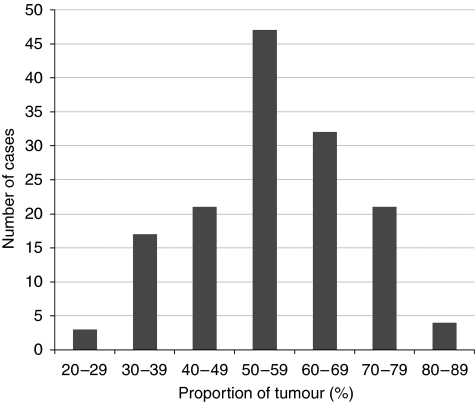
The distribution of the proportion of tumour cells across the colorectal cancer patient population.

The PoT value followed a normal distribution across the series ranging from 21.6 to 84.3% (see Figure 3). The median PoT value was 57.1% (interquartile range from 47.4 to 66.4%). The relationship between high and low PoT with clinicopathological data is shown in Table 1. There was no significant correlation between PoT and any of the clinicopathological variables, however, a low PoT was weakly related to pT stage rising from 9% in pT1 to 42% in pT4. The frequency of low PoT was higher in cancers of the rectum compared with cancers of the colon, however, this was not statistically significant (33 *vs* 21%, *P*=0.125). The PoT value was strongly inversely correlated with the proportion of stroma within the tumour (*r*=−0.913, *P*<0.0001).

### Survival analyses

**Figure 4:**
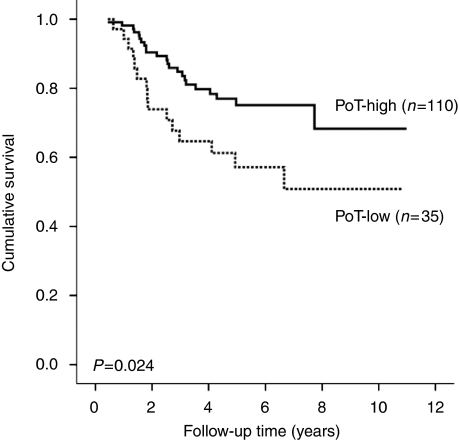
Kaplan–Meier survival curve after stratification by PoT. Hazard ratio for PoT-low=2.087 (95% confidence interval=1.088–4.003).

The results of the survival analyses for all cases are shown in Table 2. On univariate analysis, PoT-low was associated with poorer cancer-specific survival (hazard ratio (HR)=2.087, 95% confidence interval (CI)=1.088–4.003, *P*=0.024, Figure 4). This was also significant when follow-up was censored at 3 years (HR=2.878, 95% CI=1.369–6.049, *P*=0.003) and 5 years (HR=2.083, 95% CI=1.066–4.072, *P*=0.028). Multivariate analysis confirmed that PoT-low was an independent poor prognostic marker when the model was adjusted for age, pT stage, pN stage and extramural vascular invasion (*P*=0.017).

Subgroup analyses by tumour location demonstrated that PoT is a significant prognostic factor in colonic cancers (HR=2.474, 95% CI=1.132–5.408, *P*=0.019) but not in rectal cancers (HR=1.693, 95% CI=0.516–5.562, *P*=0.380). This result was confirmed on multivariate analysis for both colonic (HR=2.703, 95% CI=1.183–6.175, *P*=0.018) and rectal cancers (HR=1.610, 95% CI=0.471–5.505, *P*=0.448). Table 3 shows the survival analyses according to the TNM stage of disease. The prognostic effect of PoT remained in TNM stage III disease on both univariate (HR=3.480, 95% CI=1.325–9.136, *P*=0.007) and multivariate analysis (HR=3.121, 95% CI=1.091–8.929, *P*=0.034).

## Discussion

Malignant tumours such as CRC are supported by a rich network of stroma that undergoes varying degrees of modification after epithelial cell invasion including desmoplasia (Hewitt *et al*, 1993), angiogenesis and inflammatory cell infiltration (De Wever and Mareel, 2003). The composition of tumours varies dramatically among patients and has previously been linked to differential survival in the breast (Baak *et al*, 1985), lung (Nakajima, 1991; Maeshima *et al*, 2002; das Neves Pereira *et al*, 2004), skin (Breuninger *et al*, 1997), prostate cancer (Yanagisawa *et al*, 2007) and CRC (Halvorsen and Seim, 1989; Shepherd *et al*, 1997; Sis *et al*, 2005; Ngan *et al*, 2007; Tsujino *et al*, 2007). Most of these studies demonstrate that a greater proportion of stroma or an exaggerated desmoplastic response is associated with poorer patient outcomes (Jass, 1986; Roncucci *et al*, 1996). Studies in human colon cancer cell lines have shown that organ-specific fibroblasts can directly influence the ability of the tumour cells to invade through the production of factors such as collagenases (Fabra *et al*, 1992). Molecular studies have shown that specific chromosomal aberrations are related to the proportion of tumour in CRC and therefore may affect tumour–stroma interactions (Fijneman *et al*, 2007). Quantitative methods to evaluate desmoplasia including computer-assisted image analysis have already been shown to improve reproducibility of the assessment and have confirmed a relationship to survival (Sis *et al*, 2005; Tsujino *et al*, 2007).

Not surprisingly, our study demonstrated that the proportion of viable tumour cells within a tumour is strongly correlated to the proportion of stroma. Using quantitative point counting on virtual tissue sections, we have shown that a low proportion of malignant epithelial cells within a given CRC is independently associated with worse cancer-specific survival. The prognostic effect of PoT seemed to be more significant in colonic cancers and TNM stage III disease than rectal cancers and TNM stages I and II, although due to the small numbers involved in these subgroup analyses the results must be viewed with caution and need to be confirmed in a larger series of cases.

There are several theories as to why a low PoT (and therefore high proportion of stroma) within a tumour may infer a poor prognosis. First, it has been hypothesised that tumours with a greater proportion of reactive stroma are able to produce more growth factors thus increasing the overall tumour burden (De Wever and Mareel, 2003). Second, it has been suggested that the relative amount of desmoplastic fibrosis may have a role in reducing the accessibility of tumours to the immune response (Kouniavsky *et al*, 2002) by encapsulating the malignant cells and preventing their destruction (Liotta *et al*, 1983). However, one must consider that PoT may reflect the stage of disease, and our study suggests a weak correlation between PoT-low and pT stage, which is in concordance with one previous study in which low PoT was seen in 8% of stage I patients and in up to 69% of stage III patients (Mesker *et al*, 2007).

Morphometrical analysis enables accurate quantification of various tissue components when compared with qualitative systems. The traditional method of using optical graticules on conventional glass slides has limited flexibility. Using software that can insert any number of sampling points within a systematic grid onto a virtual slide (Treanor *et al*, 2008), scores can be entered and saved as an electronic file for further analysis. Although we have used primary resection material in this study, analysis of stromal grade has been shown to be prognostic on biopsy material from the prostate (Yanagisawa *et al*, 2007) and our technique could potentially be used on pre-operative biopsies of CRC.

We and others have observed significant heterogeneity in the PoT within individual tumours (Mesker *et al*, 2007). In this study, we selected an area at the luminal surface to allow future investigation to determine whether the results may be extrapolated to diagnostic biopsy material. It is possible that different results may be acquired if the measurements were performed in different areas of the tumour, for example, the centre or the advancing edge. In breast cancer, some studies have shown a better prognosis with higher PoT at the tumour periphery (Baak *et al*, 1985), whereas others have suggested an inverse relationship when assessing the whole tumour (Tanaka *et al*, 2004).

Much of the recent research into optimising CRC patient management has focussed on identifying (i) prognostic markers that allow us to determine which patients may benefit from adjuvant therapy and (ii) predictive markers which predict the response of individual patients to specific therapeutic regimens. The identification of patients with a poor prognosis based on an estimate of PoT obtained through simple, relatively inexpensive morphometrical measurements could easily be transferred into routine diagnostic practice according to our own experience. On the basis of the results of our present study, we hypothesise that patients with PoT-low may be more likely to respond to agents directed at inhibiting the crosstalk between stromal and epithelial cells, an area that clearly warrants further investigations.

## Figures and Tables

**Table 1:** Clinicopathological data and its association with the proportion of PoT

			**Proportion of tumour**				
	**All cases**		**High**		**Low**		
	***n***	**%**	***n***	**%**	***n***	***%***	***P*-value**
*Gender*							
Male	58	40	44	40	14	40	1.000
Female	87	60	66	60	21	60	
							
*Tumour location*							
Colon	102	70	81	74	21	60	0.125
Rectum	43	30	29	26	14	40	
							
*Adjuvant treatment*							
Yes	21	15	93	85	31	89	0.557
No	124	86	17	15	4	11	
							
*Depth of invasion (pT)*							
pT1	11	8	10	9	1	3	0.064
pT2	27	19	22	20	5	14	
pT3	95	66	71	65	24	69	
pT4	12	8	7	6	5	14	
							
*Lymph node status (pN)*							
pN0	97	67	75	68	22	63	0.650
pN1	30	21	21	19	9	26	
pN2	18	12	14	13	4	11	
							
*Distant metastasis (pM)*							
pM0	143	99	109	99	34	97	0.391
pM1	2	1	1	1	1	3	
							
*TNM stage*							
I	33	23	27	25	6	17	0.294
II	62	43	47	43	15	43	
III	48	33	35	32	13	37	
IV	2	1	1	1	1	3	
							
*Extramural vascular invasion*							
No	140	97	106	96	34	97	0.826
Yes	5	3	4	4	1	3	

Abbreviations: PoT=proportion of tumour; TNM=tumour, node, metastasis.

Staging data was obtained using TNM, 5th edition (Sobin and Wittekind, 1997, pp 66–69).

Clinicopathological data and its association with the proportion of tumour (PoT) which has been dichotomised into PoT-high (>47% of tumour cells) and PoT-low (⩽47% of tumour cells).

**Table 2:** Univariate survival data obtained with the log-rank test

	**Univariate data**			**Multivariate data**
	**Chi-square**	**Df**	***P*-value**	***P*-value**
Age (per 10-year increase)	11.824	4	0.019	0.019
Gender	1.247	1	0.264	—
Tumour location	0.061	1	0.805	—
Adjuvant treatment given	0.783	1	0.376	—
Depth of invasion (pT)	20.974	3	<0.0001	0.070
Lymph node status (pN)	6.283	2	0.043	0.011
Distant metastasis (pM)	0.336	1	0.562	—
Extramural vascular invasion	5.076	1	0.024	0.016
Proportion of tumour	5.129	1	0.024	0.017

Abbreviation: Df=degrees of freedom.

Univariate survival data obtained with the log rank test for the various clinicopathological variables, along with the relevant multivariate *P*-values obtained using a Cox proportional hazards regression analysis.

**Table 3:** Univariate survival data obtained with the log rank test according to TNM stage for PoT-high *vs* PoT-low

			**Univariate data**			**Multivariate data**
	***n* (%)**	**Median PoT (IQR)**	**Chi-square**	**Df**	***P*-value**	***P*-value**
TNM stage I	33 (23)	57 (51–64)	0.116	1	0.734	0.554
TNM stage II	62 (43)	58 (50–68)	1.013	1	0.314	0.230
TNM stage III	48 (33)	55 (45–68)	7.240	1	0.007	0.034

Abbreviations: Df=degree of freedom; IQR=interquartile range; PoT=proportion of tumour; TNM=tumour, node, metastasis classification.

Also shown are the multivariate *P*-values obtained using a Cox proportional hazards regression analysis (adjusted for age and extramural vascular invasion). TNM stage IV is not included as only two cases fell into this category.
